# Supplementary material for: Safety and effectiveness of 8 weeks of Glecaprevir/Pibrentasvir in challenging HCV patients: Italian data from the CREST study
Source: PLoS One. 2023 Feb 2;18(2):e0280165. doi: 10.1371/journal.pone.0280165 (PMC9894491; doi:10.1371/journal.pone.0280165)
Supplement: S1 Table — (DOCX) [file pone.0280165.s001.docx]

### S1 Table. Concomitant medications and their expected interaction with glecaprevir/pibrentasvir

| \| **Concomitant medications^a^** \| **Patients n=26** \| **Expected interaction with G/P^b^** \| \| --- \| --- \| --- \| \| Acetylsalicylic acid \| 5 (19.2) \| No interaction expected \| \| Bisoprolol \| 4 (15.4) \| No interaction expected \| \| Metformin \| 4 (15.4) \| No interaction expected \| \| Pantoprazole \| 3 (11.5) \| Potential weak interaction \| \| Atenolol \| 2 (7.7) \| No interaction expected \| \| Colecalciferol \| 2 (7.7) \| No interaction expected \| \| Furosemide \| 2 (7.7) \| No interaction expected \| \| Levothyroxine \| 2 (7.7) \| No interaction expected \| \| Methadone \| 2 (7.7) \| No interaction expected \| \| Perindopril \| 2 (7.7) \| No interaction expected \| \| Perindopril arg/amlodipine fisher \| 2 (7.7) \| No interaction expected \| \| Propranolol \| 2 (7.7) \| No interaction expected \| \| Ramipril \| 2 (7.7) \| No interaction expected \| \| Valsartan \| 2 (7.7) \| No interaction expected \| \| Omeprazole \| 2 (7.7) \| Potential weak interaction \| \| Telmisartan \| 2 (7.7) \| Potential interaction \| \| Other coadministered medications^c^ \|  \|  \| \| No interaction expected \| 15 (57.7) \|  \| \| Potential interaction expected \| 8 (30.8) \|  \| \| Potential weak interaction expected \| 4 (15.4) \|  \| \| Suggested no coadministration \| 2^d^ (7.7) \|  \| \| Data are n (%). \| \| \| \| ^a^Multiple selection was allowed in the case report form for this variable, so aggregate percentage may exceed 100%; ^b^Information gathered from the University of Liverpool HEP Drug Interactions Checker, available at: www.hep-druginteractions.org/checker; ^c^All the medications with n=1 each have been summarized and grouped by expected interaction; ^d^Atorvastatin and simvastatin.  *G/P* glecaprevir/pibrentasvir. \| \| \| |
| --- | --- | --- | --- | --- | --- | --- | --- | --- | --- | --- | --- | --- | --- | --- | --- | --- | --- | --- | --- | --- | --- | --- | --- | --- | --- | --- | --- | --- | --- | --- | --- | --- | --- | --- | --- | --- | --- | --- | --- | --- | --- | --- | --- | --- | --- | --- | --- | --- | --- | --- | --- | --- | --- | --- | --- | --- | --- | --- | --- | --- | --- | --- | --- | --- | --- | --- | --- | --- | --- | --- | --- | --- |
