# Supplementary material for: Safety and effectiveness of 8 weeks of Glecaprevir/Pibrentasvir in challenging HCV patients: Italian data from the CREST study
Source: PLoS One. 2023 Feb 2;18(2):e0280165. doi: 10.1371/journal.pone.0280165 (PMC9894491; doi:10.1371/journal.pone.0280165)
Supplement: Attachment — Submitted filename: Response to Reviewers.docx [file pone.0280165.s002.docx]

**Responses to Journal Comments**

**Manuscript ID:** PONE-D-22-27602

**Safety and effectiveness of 8 weeks of Glecaprevir/Pibrentasvir in challenging HCV patients: Italian data from the CREST Study**

Alessio Aghemo, Marcello Persico, Roberta D’Ambrosio, Massimo Andreoni, Erica Villa, Abhi Bhagat, Valentina Gallinaro, Giuliana Gualberti, Rocco Cosimo Damiano Merolla, Antonio Gasbarrini.

Please note that page numbers refer to pages in the TRACKED version of the manuscript.

# Journal requirements

## Requirement #1:

Please ensure that your manuscript meets PLOS ONE's style requirements, including those for file naming. The PLOS ONE style templates can be found at

<https://journals.plos.org/plosone/s/file?id=wjVg/PLOSOne_formatting_sample_main_body.pdf> and <https://journals.plos.org/plosone/s/file?id=ba62/PLOSOne_formatting_sample_title_authors_affiliations.pdf>

**Response:** We confirm that we have checked that the manuscript meets the PLOS ONE’s style requirements.

## Requirement #2:

We noticed you have some minor occurrence of overlapping text with the following previous publication(s), which needs to be addressed: <https://pubmed.ncbi.nlm.nih.gov/35543964/>.

In your revision ensure you cite all your sources (including your own works), and quote or rephrase any duplicated text outside the methods section. Further consideration is dependent on these concerns being addressed.

**Response:** Thank you for this comment. We confirm that we have revised the manuscript and rephrased any occurrence of duplicated text.

## Requirement #3:

Please provide additional details regarding participant consent. In the ethics statement in the Methods and online submission information, please ensure that you have specified (1) whether consent was informed and (2) what type you obtained (for instance, written or verbal, and if verbal, how it was documented and witnessed). If your study included minors, state whether you obtained consent from parents or guardians. If the need for consent was waived by the ethics committee, please include this information.

**Response:** We thank the reviewers for this comment, the study was granted an exemption from obtaining informed patient consent which was approved by each of the institutional ethics committees for the sites in Italy. We have added this to the methods section (Lines 106–108). Although patients younger than 18 years old were allowed, our study did not enroll any. The split of the enrolled patients by age group was:

| **Age at treatment initiation, years** | **n (%)** |
| --- | --- |
| 12-17 | 0 (0%) |
| 18-89 | 42 (100%) |
| >=90 | 0 (0%) |

## Requirement #4:

We note that the grant information you provided in the ‘Funding Information’ and ‘Financial Disclosure’ sections do not match.

When you resubmit, please ensure that you provide the correct grant numbers for the awards you received for your study in the ‘Funding Information’ section.

**Response:** We confirm that we have updated the funding information. The study was funded by AbbVie, so no grant number is available.

## Requirement #5:

Thank you for stating the following in the Competing Interests section:

“Aghemo A: Grant support by AbbVie and Gilead; Advisory board for Alfasigma, Gilead, Intercept, MSD, Mylan, and Sobi.

Persico M: Consultant and speaker for AbbVie, Gilead, and MSD.

D’Ambrosio R: Advisory Board: AbbVie, Gilead, Takeda; Speaking and teaching: AbbVie, Gilead, MSD; Research support: Gilead

Andreoni M: Board membership for AbbVie, Gilead, Merck, and ViiV; grant from Merck; and speaker for BMS, Gilead, and Janssen.

Villa E: Nothing to disclose.

Bhagat A, Gallinaro V, Gualberti G, and Merolla RCD: Employees of AbbVie and may hold stock/options.

Gasbarrini A: Consultant for AbbVie, Actial, Alfasigma, Eisai, Gilead, MSD, Sandoz, Sanofi, and Takeda.”

Please confirm that this does not alter your adherence to all PLOS ONE policies on sharing data and materials, by including the following statement: "This does not alter our adherence to PLOS ONE policies on sharing data and materials.” (as detailed online in our guide for authors <http://journals.plos.org/plosone/s/competing-interests>). If there are restrictions on sharing of data and/or materials, please state these. Please note that we cannot proceed with consideration of your article until this information has been declared.

Please include your updated Competing Interests statement in your cover letter; we will change the online submission form on your behalf.

**Response:** We confirm that we have included the statement "This does not alter our adherence to PLOS ONE policies on sharing data and materials” at the end of the competing interests section. As requested, the updated competing interests section has been included in the cover letter.

## Requirement #6:

In your Data Availability statement, you have not specified where the minimal data set underlying the results described in your manuscript can be found. PLOS defines a study's minimal data set as the underlying data used to reach the conclusions drawn in the manuscript and any additional data required to replicate the reported study findings in their entirety. All PLOS journals require that the minimal data set be made fully available. For more information about our data policy, please see <http://journals.plos.org/plosone/s/data-availability>.

Upon re-submitting your revised manuscript, please upload your study’s minimal underlying data set as either Supporting Information files or to a stable, public repository and include the relevant URLs, DOIs, or accession numbers within your revised cover letter. For a list of acceptable repositories, please see <http://journals.plos.org/plosone/s/data-availability#loc-recommended-repositories>. Any potentially identifying patient information must be fully anonymized.

Important: If there are ethical or legal restrictions to sharing your data publicly, please explain these restrictions in detail. Please see our guidelines for more information on what we consider unacceptable restrictions to publicly sharing data: <http://journals.plos.org/plosone/s/data-availability#loc-unacceptable-data-access-restrictions>. Note that it is not acceptable for the authors to be the sole named individuals responsible for ensuring data access.

We will update your Data Availability statement to reflect the information you provide in your cover letter.

**Response:** All relevant data are within the manuscript, except for the raw demographic and clinical information. Demographic and clinical information cannot be shared publicly as it potentially contains sensitive information. However, as mentioned in the Data Availability statement (uploaded in the submission website), the clinical trial data can be requested by any qualified researcher, and will be provided following review and approval of a research proposal.

## Requirement #7:

Please review your reference list to ensure that it is complete and correct. If you have cited papers that have been retracted, please include the rationale for doing so in the manuscript text, or remove these references and replace them with relevant current references. Any changes to the reference list should be mentioned in the rebuttal letter that accompanies your revised manuscript. If you need to cite a retracted article, indicate the article’s retracted status in the References list and also include a citation and full reference for the retraction notice.

**Response:** We confirm that the reference list is complete and correct.

# Reviewer #1:

## General comments:

Thank you for the opportunity to review this article.

In this work, the authors evaluated the real-world safety and effectiveness of 8-week glecaprevir/pibrentasvir therapy in Italian treatment-naïve patients with compensated cirrhosis, analysing data in certain difficult to treat subgroups of interest.

The paper is well written and adds new important data to the real-world experience with the drug, specifically in the Italian context.

## Comment #1:

I think that study population is not big enough to sustain a robust message on the efficacy and safety of the drug primarily in GT3 patients (7/42) and in patients with signs of portal hypertension. I think that key messages of conclusions (abstract and text) should be re-modulate shedding light mainly on the core message of the work (real-world safety and effectiveness of short course treatment in cirrhotic patients) and on the need to extend the real-world cohort (Italian and beyond) to confirm the preliminary data that appear to confirm safety and effectiveness of the drug also in special subgroups (GT3, portal hypertension).

**Response:** We thank the reviewer for raising this query. We have amended the conclusions in the abstract and in the main text to incorporate your suggestions (lines 54–58, and 267–276)

## Comment #2:

Moreover, Table 3 does not add as much to the work. At best, it could be considered as supplementary material.

**Response:** We thank the reviewer for this comment. We have moved Table 3 to supplementary material (S1 Table).

# Reviewer #2:

## General comments:

Dear Editor,

Aghemo et al wrote a paper on a sub-analysis of a large retrospective observational non interventional multicentric study conducted in six countries worldwide to evaluate in a real-life setting the efficacy and safety of the Glecaprevir/Pibrentasvir direct antiviral agents (DAA) combination for the treatment of HCV-related compensated cirrhosis (CC). The present paper is focused on the Italian sub-cohort of patients included. Even if, nowadays, there are several reports on the efficacy of such drugs in real life settings, some concerns (mostly preconcepts) remain on the safety of NS3-containing DAA combinations, particularly in regards of safety and DDI (drug-drug interactions). For these reasons, every report on the real-life performance of such drugs is welcome and has a potential high interest to the readership of your journal. The paper reports high efficacy and safety both on per-protocol and intention-to-treat analysis in the 42 patients with CC included.

Moreover, the paper is lean and well-written in English.

## Comment #1:

Introduction section, line 69 and beyond: “A probabilistic model estimated that in January 2020 there were more than 400,000 individuals with chronic HCV infection in Italy, with approximately 73% asymptomatic and potentially undiagnosed or unlinked to care”.

There are several reports published in Liver International journal earlier this year that somewhat seem disavowing this projection, please briefly comment this fact and cite these papers (10.1111/liv.15314 ; 10.1111/liv.15273 ; 10.1111/liv.15316; 10.1111/liv.15375).

**Response:** We are grateful to the reviewer for bringing this to our attention. We have included further details and the additional references in the paragraph from lines 77 to 79.

## Comment #2:

Conclusions section, line 242 and beyond: it could be useful to the aims of the paper to insert a brief sentence in the conclusions on the fact that, with these types of treatment, it could have to be pointed out that the so-called "difficult-to-treat" patients are definitely no longer to be considered in this way.

**Response:** We thank the reviewer for this comment. We have incorporated your suggestion in lines 271–272.
